# Wandsworth Guardians and Administrative Reform

**Published:** 1913-05-10

**Authors:** C. Thackray Parsons

**Affiliations:** Fulham Infirmary, St. Dunstan's Road, Hammersmith, W.


### The Editor's Letter-Box.

### Wandsworth Guardians and Administrative Reform.

*To the Editor of* The Hospital.

Sir,—The paper on the "Wandsworth Guardians and Administrative Reform" in last week's Hospital deals with a matter of great interest to the indoor Poor Law medical service. The administrative control of the two infirmaries of the Union is now under consideration by the Board of Guardians, who appear, judging by a recent speech of the Chairman, to favour a scheme similar to that suggested by the writer of the article. There are, however, grave objections to the proposed alterations. The four institutions, the administration of which it is proposed to place under one head, are scattered over a wide area, so that a considerable amount of time would be taken up in travelling between them. They are buildings of considerable size—the workhouses contain 1,168 beds, the St. James' Infirmary 612 beds, the St. John's Infirmary 652 beds, and the Tooting Homes 702 beds. It is obvious that if one medical man is to undertake the administration of all of these institutions the whole of his time will be fully occupied with administrative work. He will have no time to perform surgical operations himself or to consult on difficult cases with his subordinate medical officers. The whole of the medical and surgical work will have to be left to the junior medical staff. Another objection is that the proposed scheme would reduce the number of medical superintendents in London by one. One of the great drawbacks to the indoor medical service is that there are so few opportunities of advancement for the junior medical officers. It is therefore difficult to get able and experienced men to remain in the service. To make the service more attractive to the best men it is necessary to increase and not to reduce its prizes. In so far as the scheme improves the position of the assistant medical superintendent it deserves support, but the desirability of the retention of the post of medical superintendent at both infirmaries remains. The work in these two large institutions requires there should be at least two senior and experienced medical men in each of them. The classification of the cases and the avoidance of friction, upon which stress is laid, and rightly laid, in the paper, could be obtained quite as well by making the St. James' Infirmary the receiving institution for all cases of sickness, and leaving it to the medical superintendent of that institution to allocate the different cases amongst the four institutions in accordance with a general scheme based upon the special facilities for treatment afforded by each. —I am, Sir, yours faithfully,

C. Thackray Parsons.

Fulham Infirmary, St. Dunstan's Road,

